# Practical Notes on Diagnosis and Treatment

**Published:** 1908-06-13

**Authors:** 


### Practical Notes on Diagnosis and Treatment.

**Humanised Milk.**

Mr. P. E. Barber says that the nearest approximation to human milk is secured by the following: Whey 18 drachms, cow's milk 6 drachms, cream 1 drachm, and sugar 1 teaspoonful.

**Hutchinson's Teeth.**

According to a recent French writer, Hutchinson's teeth may, in cases of hereditary syphilis, be seen in the milk teeth as well as in those of the second dentition.

**Sterilised Foods.**

All these advertised sterilised foods are bad for children's teeth; and if a child must be brought up on cow's milk, the milk should not be dealt with in any way likely to destroy or change that living something which is essential for the healthy development of the child.—*Mr. Edmund Owen.*

**To Remove Tattoo Marks.**

Cover with a paste made of salicylic acid and glycerine, and keep this applied for several days by means of a compress. After removal apply a further layer of the paste. This is usually successful, but sometimes a third application is required.—*Dr. W. P. Thomas.*

**Gonorrhœal Rheumatism.**

Major F. J. W. Porter, R.A.M.C., reports six cases of severe gonorrhœal rheumatism in which complete recovery followed the use of anti-gonococcic serum. The injections (25 c.c.) are advised as soon as symptoms of the arthritis appear, and in severe cases should be repeated daily for five or six days consecutively.

**Cancer of the Rectum.**

If a patient of middle age is in the habit of going to stool half a dozen or more times a day, and yet only passes a proper motion once, and at the other times passes a dark discharge, you may be practically certain he has cancer. Rectal examination will usually confirm this conclusion, though occasionally the disease is beyond reach of the finger.

**Interstitial Keratitis in Acquired Syphilis.**

According to Mr. Herbert Fisher, interstitial keratitis in acquired syphilis is more common than is generally supposed. He regards the keratitis as a tertiary manifestation, and says that, while generally identical in clinical appearance with that occurring in the inherited disease, only one eye is usually attacked, and frequently the infiltration is limited to a part of the cornea.

**Stomatitis.**

Of internal remedies none equals a combination of iron and chlorate of potash, which may be safely given at all ages. Potassii chloratis gr. x.; tinct. ferri perchlor., ♏x.; glycerini, ♏xx.; aq. destill. ad ℥ss. may be taken in water four times a day.—*Prof. Whitla.*

**Hypodermic Alimentation.**

In emaciated patients, wherever food cannot be given by the mouth, subcutaneous injections of olive oil may be used to promote nutrition. As much as 160 grammes of the oil may be introduced in the course of twenty-four hours. Benefit from this procedure has been noted in tuberculous laryngitis, in phthisis pulmonalis, and in enteric fever.

**Tetany and Convulsions.**

Dr. F. C. Eve records the case of an infant with severe indiopathic tetany in which, after various kinds of treatment had proved unsuccessful, lumbar puncture was followed by early relief and permanent cure. An ounce of the cerebro-spinal fluid was withdrawn. The same treatment has been successfully practised in infantile convulsions and epilepsy.

**Pernicious Anæmia and Gastric Cancer.**

These two conditions are not always easily distinguished. Careful examination of the blood is usually, but not always, of great service. It may be remembered, too, that in most cases of pernicious anæmia, though there is profound anæmia, wasting is anything but a prominent feature. In cancer the anæmia and wasting advance *pari passu.*

**Prickly Heat.**

Dr. Pearse recommends the anointing of the body with oil as a certain method of keeping off this irritating condition. He uses a mixture of olive oil and lanoline in the proportions of eight to one, with some ol. rosæ. The very faint smell of this mixture disappears when it is rubbed into the skin. Soap should be avoided.

**Healthy Ovaries and Diseased Tubes.**

It is surprising how frequently healthy or comparatively healthy ovaries may be associated with marked tubal infection. In a series of thirty-two cases in only three were both ovaries so diseased as to require removal, in eleven one ovary had to be removed, and in eighteen neither ovary had to be removed. This is an important point, as it makes a great difference to the subsequent comfort of the patient if even a portion of an ovary can be left.—*Dr. Jellett.*

